# Impact of a narrative medicine programme on healthcare providers’ empathy scores over time

**DOI:** 10.1186/s12909-017-0952-x

**Published:** 2017-07-05

**Authors:** Po-Jui Chen, Chien-Da Huang, San-Jou Yeh

**Affiliations:** 1 Department of Medicine, Chang Gung University, College of Medicine, Taipei, Taiwan; 2 Chang Gung Medical Education Research Center (CG-MERC), Department of Thoracic Medicine, Chang Gung Memorial Hospital, 199 Tun Hua N. Rd, Taipei, Taiwan; 3 Department of Medical Education, Chang Gung University, College of Medicine, Taipei, Taiwan; 4 Thoracic Medicine, Chang Gung University, College of Medicine, Taipei, Taiwan; 5 Cardiology, Chang Gung Memorial Hospital, Chang Gung University, College of Medicine, Taipei, Taiwan

**Keywords:** Narrative medicine, Empathy, Educational programme, Gender

## Abstract

**Background:**

The cultivation of empathy for healthcare providers is an important issue in medical education. Narrative medicine (NM) has been shown to foster empathy. To our knowledge, there has been no research that examines whether a NM programme affects multi-professional healthcare providers’ empathy. Our study aims to fill this gap by investigating whether a NM programme effects multi-professional healthcare providers’ empathy.

**Methods:**

A pre-post questionnaire method was used.142 participants (*n* = 122 females) who attended the NM programme were divided into single (*n* = 58) and team groups (*n* = 84) on the basis of inter-professional education during a period of 2 months. Perceptions of the NM programme were collected using our developed questionnaire. Empathy levels were measured using the Chinese version of Jefferson Scale of Empathy - Healthcare Providers Version (JSE-HP) – at three time points: prior to (Time 1), immediately after (T2), and 1.5 years (T3) after the programme.

**Results:**

Participants’ perceptions about the NM programme (*n* = 116; *n* = 96 females) suggested an in enhancement of empathy (90.5%). Empathy scores via the JSE-HP increased after the NM programme (T1 mean 111.05, T2 mean 116.19) and were sustainable for 1.5 years (T3 mean 116.04) for all participants (*F*(2297) = 3.74, *p* < .025). A main effect of gender on empathy scores was found (*F*(1298) = 5.33, *p* < .022). No significant effect of gender over time was found but there was a trend that showed females increasing empathy scores at T2, sustaining at T3, but males demonstrating a slow rise in empathy scores over time.

**Conclusions:**

NM programme as an educational tool for empathy is feasible. However, further research is needed to examine gender difference as it might be that males and females respond differently to a NM programme intervention.

**Electronic supplementary material:**

The online version of this article (doi:10.1186/s12909-017-0952-x) contains supplementary material, which is available to authorized users.

## Background

In today’s society, the relationship between doctors and patients is changing and the levels of trust and understanding between patients and physician appears to be weakened [1–3]. Doctors’ opinions are not as unquestionable as in the past. Some patients may challenge doctors based on the medical information they read on the internet and even prefer folk remedies gleaned from others, rather than doctors’ diagnoses [4]. Empathy is a key element of patient–physician communication [5]. According to the definition proposed by Hojat, physician empathy is a multidimensional concept involving cognitive and affective domains. The former involves the ability to understand another person’s inner experiences and feelings alongside a capability to view the outside world from the other person’s perspective. The latter involves the capacity to enter into or join the experiences and feelings of another person [5, 6]. Therefore, these concepts help physicians to build a trustful and interdependent relationship with their patients [1] and may benefit the outcome of medical procedures or treatments [7]. In a factor analysis study, 52% of the variance in patients’ ratings of satisfaction with their medical care was accounted for by the physicians’ level of interpersonal warmth and respect [8], an affective capacity to be sensitive to and concerned for another person; both of which are among the features of the affective domain of physician empathy [5, 9]. A positive relationship between physicians’ empathy and patients’ clinical outcomes was also confirmed, suggesting that physicians’ empathy is an important factor associated with clinical competence and patient outcomes [7]. However, the cultivation of the sense of empathy is a long-term effort, the development of which is still not clear [10].

Despite the capacity for empathy being affected by innate characteristics, many people can benefit from exercises and techniques designed to foster empathy [11–13]. For example, narrative writing has been shown to effectively foster empathy in post-graduate year one (PGY1) psychiatric residents working with severely and persistently mentally ill patients [13].

The phrase “Narrative medicine” was first used in 2000 by Rita Charon to refer to clinical practice fortified by narrative competence, that is, the ability to acknowledge, absorb, interpret, and act on the stories and plights of others. In other words, it is a kind of medical performance with narrative skill and has been offered as a model for humanism, compassion and effective medical practice [14, 15]. Narrative medicine has therefore been posited as a way for physicians to understand the personal connections between themselves and their patients [14]. Using this approach it has been argued that physicians can reach out and link with their patients through narrative competence, further understanding their own personal journeys through medicine, recognizing their empathy with and responsibilities toward other healthcare professionals, and achieve meaningful discussions with patients about their healthcare [7, 16, 17]. Narrative medicine is also thought to help physicians recognize, interpret, and be moved to action by the problems of others; encouraging them to develop confidence and competence while identifying the conflicts they face [15]. Furthermore, while patients need a physician to diagnose and treat their illness, a physician with empathy who understands their suffering and who can accompany them through their illness journey is also equally important [14]. The concept of narrative medicine has therefore been suggested as a way of enabling a physician to satisfy patients in this respect [15]. It is anticipated that when patients feel satisfied, trust between them and their physicians can develop thereby facilitating patients’ openness to physicians’ advice [2, 3, 18]. Thus, the development of empathy in medical workers, paramedical staff and even medical students is of crucial importance. As such, the impact of a narrative medicine course on empathy cultivation for healthcare providers is therefore an interesting and important issue.

To our knowledge, there has been no research that examines whether a narrative medicine programme can positively effect multi-professional healthcare providers’ empathy, although previous research has already suggested that guided narrative writing designed to promote reflective thinking can help practicing physicians to explore reflection and might enhance empathy [19]. This study therefore fills a gap in current literature with the aim of investigating if and how a narrative medicine programme as an educational programme affects the empathy of clinicians. We specifically asked the following research question: RQ1: Does a narrative medicine programme increase healthcare providers’ empathy scores over time?; RQ2: Are there any differences in empathy scores according to the gender of the learner?

## Methods

### Study participants

A total of 142 participants, *n* = 122 females, comprising: physicians, traditional Chinese physicians, dentists, nurses and paramedical workers including pharmacists, medical technologists, physical therapists, respiratory therapists, and nutritionists of the largest teaching hospital in Taiwan volunteered to attend the narrative medicine programme. Participants were divided into single (*n* = 58, 50 females) and team groups (*n* = 84, 72 females) for a period of 2 months. The team groups comprised either participants from the same healthcare profession or from two different healthcare professions. The individual participants in the single group competed by writing a narrative article regarding clinical cases, while the team group performed a drama about patients’ suffering in the aspects of society, humanism and ethics. The theoretic basis for dividing participants was based on inter-professional education [20], an important pedagogical approach for preparing healthcare professions to provide patient care in a collaborative team environment. Significant overlaps were found between empathy, teamwork and integrative approach to patient care [21].

### Narrative medicine programme

Department of Medical Education planned the “Narrative Medicine” programme, which was a narrative medicine competition program with continuous announcement in the hospital for 2 months. It was based on a competition style. Before the narrative medicine program, the protocol for narrative writing began with a lecture explaining the theory and introducing the process. This activity was integrated as a one-hour session into the curriculum of faculty development.

Individual entries were open to all participants (irrespective of whether they participated in individual or team groups). In the single groups, participants represented clinical stories in their narrative writing. This activity was designed to enhance medical humanism sensibility through the processes of enabling participants to recognize, to interpret and to be moved to action by the problems of others. Through the act of narrative writing, participants could review their journeys across their clinical experiences: rethinking and reflecting on the stories they gathered from patients. Either real or simulated clinical cases were acceptable. The groups were required to act out their written case of the narrative medicine, in which the leader would guide the participants to be empathetic to the illness experiences of the patient in the case with emphasis on the social, cultural or ethical aspects. If the case scenario could not be presented through the acting, a prepared film could be played in sections in order to assist the presentation. The programme aims to help participants to integrate medical humanities practices into the medical environment, which they were familiar with, and to encourage medical staff from different specialties to learn and exchange knowledge from each other in order to achieve the teaching effectiveness of holistic health care.

Three experts from the relevant fields were invited as judges. For reviewing procedures and criteria (individuals), the judges would score each individual entry based on the written documents. The groups had 20 min to act out the teaching scenario based on the narrative medicine case. Reviewers scored the groups based on their case and their acting according to the items on the evaluation form.

Prizes were granted to the award recipients. The programme results were publicized on the Latest News on the official website of Department of Medical Education. The prizes and certificates were awarded publicly during the meeting of Education Support Funding Programme of the Department of Medical Education with ethics clearance.

### An excerpt of a narrative medicine case [translated from Chinese]

#### When the beginning life meets the end

*“It was an ordinary day, like any other day. Mums’ crying due to the labour pain came from the labour room from time to time. Patients who were scheduled for cesarean section surgeries were sent down from their ward one by one. Some pregnant women were waiting at the nursing station for check-up. The attending physicians came to do the ward round … after taking over the shifts, various kinds of staff (ward clerks, assistants, doctors, and nurses …) dashed to their destinations like well-trained fighter planes. A busy day started. At that time, a man was pushing a wheelchair where a pregnant mum with a big belly and painful expression on her face was seated. “My wife seems to be going to deliver.” Our colleagues approached them, asked for her child-bearing history, and led the husband to help his wife have the exam on the exam table … “Head Nurse, could you help me check the heartbeat? I couldn’t find it.” The colleague came over here from the exam room. I went to assist, but still could not hear the heartbeat, so I comforted the mum who was having labour pains by saying, “Please hold on for a second. We are asking the doctor to come here.” And then the Chief Resident and the attending physician came one after another in a hurry and they checked by ultrasound. “Indeed there’s no heartbeat!” Suddenly the exam room went silent. The mum and the husband also felt the unusual atmosphere, “What? What happened? What happened to my baby?” The attending physician replied difficultly, “Mum, the child has no heartbeat. It is gone.” … “How is this possible? I was feeling her moving this morning. She was kicking me. It’s impossible. Doctor, please do the operation right now. Do the operation and rescue the baby. Please hurry up …*” *And then I heard the crying. “Mum, she has had no heartbeat. It’s impossible to rescue her by doing the operation.” The attending physician tried to get the mum to realise that the fetus was dead in her womb. “Impossible! Daddy, how is this possible? How is this possible?” The husband seemed to understand the doctor’s explanation. “Mummy. It’s okay. It’s okay.” At that moment, we circled around the mum, held her, and comforted her. However, all the way from the exam room to the labour room, she was still having an emotional breakdown and crying very hard …”.*

### Questionnaire development

The questionnaire comprised a 10-item survey instrument administered using a 5-point Likert scale (strongly disagree to strongly agree) developed by four experts in clinical education and faculty development Additional file 1. These experts reviewed the items for content and face validity. A pilot check with faculty members was performed examining internal consistency and reliability. The questionnaire investigated two domains of participants’ perceptions: perceptions about the narrative medicine programme and personal attitudes about the narrative medicine progress model.

#### Perceptions about the narrative medicine programme

- Narrative medicine (NM) is helpful for reflection
- NM is helpful for enhancement of empathy
- NM is helpful for the relationship between patients and doctors
- NM is helpful for relieving my grief during medical care
- NM is essential for medical care
- NM relieves my pressure during medical care

#### Personal attitudes about the narrative medicine progress model

- I have a good overall impression on NM
- I am interested in NM
- I will tell my co-workers about the concept of NM
- I will continue with my narrative writing

### Empathy instrument and survey

The Jefferson Scale of Empathy (JSE) was developed in 2001 at Jefferson Medical College as an instrument to measure empathy in the context of medical education and patient care [5, 22, 23]. The instrument relies on the definition of empathy in the context of patient care as a predominantly cognitive attribute that involves an understanding of the patient’s experiences, concerns, and perspectives, combined with a capacity to communicate this understanding and an intention to help [24, 25]. The scale includes 20 items answered on a seven-point Likert-type scale (Strongly Agree =7, Strongly Disagree =1).

We used the Chinese version of Jefferson Scale of Empathy - Healthcare Providers Version (JSE-HP) previously published to measure the empathy of participants anonymously [26]. The participants completed the JSE three times anonymously: before the programme (*n* = 110), immediately after the programme (*n* = 100), and one and a half years after the programme (*n* = 90) as a long-term follow up of empathy change. In addition to empathy, the perceptions of the participants on the narrative medicine programme were also recorded by a questionnaire after the programme anonymously using the questionnaire described above (*n* = 116). A waiver of the requirement to obtain the written informed consent was approved. Ethical approval for this study was obtained from the Chang Gung Memorial Hospital and Chang Gung University Institutional Review Board (IRB No. 102-4138B, 103-1755B, 105-2716C).

### Statistical analysis

All data are expressed as mean values and standard error of mean (SEM) or as numeric values and percentages (%). A between groups t-test assessed differences between participants undertaking the programme in groups versus those undertraining individually. A one-way ANOVA was used to compare empathy scores over three time points (Time 1 (T1) before, Time 2 (T2) immediately after, and Time 3 (T3) 1.5 years after the programme). A between subjects ANOVA examined the main effect of gender and interaction of gender and time for empathy scores. The level of statistical significance was set at *p* < 0.05. All analyses were conducted using SPSS software (version 13.0, SPSS, Chicago, IL), and Prism 5 for Windows (version 5.03, GraphPad Software Inc., San Diego, CA).

## Results

### Participants’ perceptions about narrative medicine (table 1)

The response rate for the perceptions of narrative medicine programme questionnaire was 81.7% (116/142) immediately after the programme. Participants’ perceptions were positive (strongly agree and agree) in terms of enhancement of reflection (106/116, 91.4%), empathy (105/116, 90.5%), and patient-doctor relationships (98/116, 84.5%). Furthermore, the participants were generally willing to tell their coworkers about the concept of narrative medicine (98/116, 84.5%).

**Table 1:** Participants’ perceptions about narrative medicine (*n* = 116)

Item	Very agree and agree (n,%)	Neutral (n,%)	Very disagree and disagree (n,%)
NM is helpful for reflection	106 (91.4%)	8 (6.9%)	2 (1.7%)
NM is helpful for in enhancing empathy	105 (90.5%)	9 (7.8%)	2 (1.7%)
NM is helpful for patient-doctor relationships	98 (84.5%)	15 (12.9%)	3 (2.6%)
I will tell my coworkers about the concept of NM	98 (84.5%)	15 (12.9%)	3 (2.6%)
I have a good overall impression on NM	97 (83.6%)	17 (14.7%)	2 (1.7%)
I am interested in NM	96 (82.7%)	17 (14.7%)	3 (2.6%)
NM relieves my grief during medical care	96 (82.7%)	15 (12.9%)	5 (4.3%)
NM is essential for medical care	94 (81.0%)	19 (16.4%)	3 (2.6%)
I will continue with my narrative writing	86 (74.1%)	27 (23.3%)	3 (2.6%)
NM relieves my pressure during medical care	77 (66.4%)	26 (22.4%)	13 (11.2%)

*NM* Narrative medicine

### Overall empathy degree change of participants (table 2)

No significant difference was found between participants undertaking the narrative medicine programme as a group or as an individual. A significant effect of time was found: empathy scores increased immediately after the narrative medicine programme (T1 mean 111.05, T2 mean 116.19) and this increase was sustained (T3 mean 116.04) for one and a half years (*F*(2297) = 3.74, *p* < .025). Post hoc tests found significant changes of empathy scores for participants post-programme (T1 vs T2; SE 2.14, *p* < .017) and one and a half years follow-up (T1 vs T3; SE 2.20, *p* < .024) when compared with the pre-programme scores.

**Table 2:** Overall empathy degree change of participants

	Pre-programme (T1) (*n* = 110)	Post-programme (T2) (*n* = 100)	1.5 years follow-up (T3) (*n* = 90)	*p*
*Total*	111.1 ± 1.4	116.2 ± 1.6^*^	116.0 ± 1.6^*^	0.025

Time 1 (T1): before, Time 2 (T2): immediately after, and Time 3 (T3): 1.5 years after programme

^*^ *p* < 0.05 when compared with T1

### Changes in empathy scores: Gender difference (table 3)

A main effect of gender was found (*F*(1298) = 5.33, *p* < .022): empathy scores in females (Total: 115.1 ± 0.9, *n* = 260) was higher when compared with those in males (Total: 109.0 ± 3.1 *n* = 40). There was no significant interaction between gender and time. However, males and females demonstrated a difference in trend: female participants (T1:111.4 ± 1.5, *n* = 97; T2:117.9 ± 1.6, *n* = 85; T3:116.6 ± 1.6, *n* = 78) demonstrated greater enhancement in empathy immediately after the programme (T2: *p* < .003) which was maintained over time after one and a half years follow-up (T3: *p* < .018) when compared with pre-programme. Male participants (T1:108.6 ± 5.0, *n* = 13; T2:106.7 ± 5.3, *n* = 15; T3:112.3 ± 5.9, *n* = 12) had no immediate improvement in empathy scores, but an increase in empathy scores was identified after one and a half years.

**Table 3:** Changes in empathy scores: gender difference

	Pre-programme (T1) (*n* = 110)	Post-programme (T2) (*n* = 100)	1.5 years follow-up (T3) (*n* = 90)	Total (*n* = 330)
*Female*	111.4 ± 1.5 (*n* = 97)	117.9 ± 1.6^*^ (*n* = 85)	116.6 ± 1.6^*^ (*n* = 78)	115.1 ± 0.9^#^ (*n* = 260)
*Male*	108.6 ± 5.0 (*n* = 13)	106.7 ± 5.3 (*n* = 15)	112.3 ± 5.9 (*n* = 12)	109.0 ± 3.1 (*n* = 40)

Time 1 (T1): before, Time 2 (T2): immediately after, and Time 3 (T3): 1.5 years after programme

^*^ *p* < 0.05 when compared with T1, ^#^ *p* < 0.05 when compared with corresponding “males”

## Discussion

Our study shows that the overall empathy scores as measured by the Jefferson Scale of Empathy (JSE) increased immediately after the narrative medicine programme and was generally sustainable for at least one and a half years. Thus, not only do empathy scores not reduce, our results suggest that it may be possible to attribute this effect of positive changes in empathy scores to the introduction of our narrative medicine training into a medical training course.

Previous studies reporting the effects of general educational interventions to promote empathy have tended to be inconclusive [27], although the majority do report a positive result from targeted empathy training [27, 28]. That our targeted intervention appears to have had a positive and sustained effect is a valuable finding and supports other recent evidence using a medical student cohort [29, 30]. As such this adds to the data contradicting previous studies that demonstrate a significant downward trend in self-assessed empathy for residents in their clinical training [10].

Our study sits well with other studies utilizing a specifically narrative-based approach to facilitate empathy in an undergraduate setting which have also demonstrated an increase in empathy scores [31]; although again, findings in these studies are mixed (for example, empathy increasing according to the Balanced Emotional Empathy Scale (BEES) [32], but demonstrating no change on the Empathy Construct Rating Scale (ECRS)) [33]. Another more specific study has also shown that narrative writing effectively fosters empathy in a PGY1 psychiatric resident population working with severely and persistently mentally ill patients [13]. Having observed similar results in our study, we believe that a narrative medicine programme could indeed be an effective way to enhance empathy in physicians, medical students, and other health professionals.

Our study reveals that most of our participants held a positive attitude towards applying narrative concepts to their medical care. In some ways this is not surprising because medicine has never been without a narrative element. Medicine is an enterprise in which one human being extends help to another; it has always been grounded in life’s intersubjective domain [34, 35]. Medical practice also requires an authentic engagement between persons that is transformative for all parties involved [14]. Empathy and reflection are essential in building an effective patient-physician relationship [18]. Thus we find that participants in our study showed a high agreement rate with the statement that narrative medicine is helpful for the relationship between patients and doctors. Furthermore, the majority of participants who experienced our programme reported a willingness to disseminate the concept of narrative medicine with their co-workers and peers. This suggests that the impact of the programme could be wider than the immediate participant group.

This brings us to the question of whether the narrative medicine programme has a similar impact for all participants. We found that gender tends to be a factor in terms of empathy scores overall with females’ scores being higher than males’, although we found no significant difference of gender over time. However, on further examination, we did find that female and male participants tended to respond differently to the narrative medicine programme. Female participants showed increased scores immediately after the programme, while males had an initial small decrease in empathy scores. The empathy scores of female participants then reached a plateau over time, maintaining their level of empathy 1.5 years later. By contrast, male participants showed a different pattern, demonstrating a gradual enhancement of their empathy scores across the 1.5 years time period.

The finding that women generally tend to score higher on empathy ratings than men is consistent with the findings of other studies [5, 36, 37]. Indeed, it has been suggested that women tend to be more receptive than men to emotional signs [38]. There is also evidence that females tend to record higher scores on self-reported measures of empathy [39]. From our results, we tentatively conclude that female medical workers might respond to empathy training via narrative medicine techniques relatively quickly, and maintain the achievement over time, whereas males might need a longer time span to digest the experiences provided by a narrative medicine programme. This finding resonates with other studies that suggest females respond more to educational interventions designed to increase empathy [37, 40]. However, these studies did not include the long-term follow up as we have done, therefore, the pattern whereby males gradually catch up is not replicated in them.

### Study limitations

Our study has several limitations. First, the participants in our study are all self-selected, with a higher proportion of females than males. In contrast with the general health professional population, it could be that our participants might be more interested in, or open to, reflection and empathic communication and thus be more receptive to an empathy-focused education programme. The changing trends of empathy scores we have observed therefore may not represent that of general medical and healthcare professionals. Furthermore, due to the imbalance in male and female participants, our study findings may only be generalizable to a female population. Whilst the interaction between gender and time was not significant, had there been more male participants in our study, this trend might have achieved significance. Thus additional studies are needed to explore a larger group with a better gender-balance of participants. Furthermore, studies matching individual scores longitudinally over time are also needed to ascertain if these differences still hold. Alternatively, following participants over time using qualitative interview methods might enable us to unpack the relative impact they perceive such interventions to have over time.

Secondly, as patient-centered medical care is now a worldwide movement [41], it is important that we not only consider self-reported measures of empathy [42], but we also explore patient-perspectives of healthcare providers’ empathy. Evaluating patients’ perceptions of their healthcare providers’ empathy when receiving medical care would therefore be a valuable extension of this project.

Finally, we used the JSE to measure participants’ empathy. However, empathy has been described as a multi-dimensional construct, comprising two main domains: an affective capacity to be sensitive to and concerned for another person; and a cognitive capacity to understand and appreciate the other person’s perspective [9]. The JSE [23, 43] only measures the cognitive dimension of empathy. Furthermore, a number of the items in this tool comprise general statements about possible therapeutic benefits of empathy and as such has been criticized for being too far-removed from real-life patient interactions [44]. As such this is considered to be a drawback when using this particular scale to measure empathy.

## Conclusions

In this study, we have explored the impact of a narrative medicine programme and its effect on participants’ empathy scores according to gender. While a narrative medicine programme appears feasible as an educational program to be an empathy enhancer, such an intervention alone would not be sufficient for developing an overall effective empathy training programme for healthcare providers. However, developing effective educational strategies to enhance healthcare providers’ empathy targeted at different healthcare groups according to their gender might appear to be a necessary consideration.

## Additional file

Additional file 1: English version of questionnaire for narrative medicine. Participants’ perceptions about narrative medicine for Table S1. The results show that participants’ perceptions were positive (strongly agree and agree) in terms of enhancement of reflection (106/116, 91.4%), empathy (105/116, 90.5%), and patient-doctor relationships (98/116, 84.5%). (DOC 48 kb)

## Abbreviation

HP: Healthcare providers
IRB: Institutional Review Board
JSE: The Jefferson Scale of Empathy
NM: Narrative medicine
SEM: Standard error of mean
