# Supplementary material for: Impact of a narrative medicine programme on healthcare providers’ empathy scores over time
Source: BMC Med Educ. 2017 Jul 5;17:108. doi: 10.1186/s12909-017-0952-x (PMC5499008; doi:10.1186/s12909-017-0952-x)
Supplement: Additional file 1: — English version of questionnaire for narrative medicine. Participants’ perceptions about narrative medicine for Table S1. The results show that participants’ perceptions were positive (strongly agree and agree) in terms of enhancement of reflection (106/116, 91.4%), empathy (105/116, 90.5%), and patient-doctor relationships (98/116, 84.5%). (DOC 48 kb) [file 12909_2017_952_MOESM1_ESM.doc]

**Questionnaire for Narrative Medicine**

Thank you very much for your participation for narrative medicine programme. Please rate the following items anonymously.

**Fill in the basic information**

1. Gender: □Female □Male

2. Seniority: □≤5 years □6 -9 years □ ≥10 years

3. Participant group: □ Single group □ Team group

4. Participants position：□Western Medicine doctor □Traditional Chinese Medicine doctor □Dentist □Pharmacist □Medical technologist □Nurse □Radiology Technologist □Physical therapists □Respiratory therapists □Nutritionists □Clinical psychologist

Date：

**Please rate the following items on a 5-point Likert scale (strongly disagree to strongly agree)**

| Items | Very agree | Agree | Neutral | Disagree | Very disagree |
| --- | --- | --- | --- | --- | --- |
| 1. **I am interested in NM** |  |  |  |  |  |
| 2. **NM is essential for medical care** |  |  |  |  |  |
| 3. **NM is helpful for patient-doctor relationships** |  |  |  |  |  |
| 4. **NM is helpful for reflection** |  |  |  |  |  |
| 5. **NM relieves my grief during medical care** |  |  |  |  |  |
| 6. **NM relieves my pressure during medical care** |  |  |  |  |  |
| 7 **NM is helpful for in enhancing empathy** |  |  |  |  |  |
| 8. **I will continue with my narrative writing** |  |  |  |  |  |
| 9. **I will tell my coworkers about the concept of NM** |  |  |  |  |  |
| 10. **I have a good overall impression on NM** |  |  |  |  |  |

The questionnaire is finished. Thank you for your rating.
